# Tetrandrine Inhibits Titanium Particle-Induced Inflammatory Osteolysis through the Nuclear Factor-*κ*B Pathway

**DOI:** 10.1155/2020/1926947

**Published:** 2020-11-27

**Authors:** Zige Liu, Yan Li, Fengying Guo, Chen Zhang, Guorui Song, Jiahao Yang, Desheng Chen

**Affiliations:** ^1^Department of Orthopedic Surgery, General Hospital of Ningxia Medical University, Yinchuan 750004, China; ^2^School of Clinical Medicine, Shimane University, Shimane 693-8501, Japan; ^3^School of Basic Medical Sciences, Ningxia Medical University, Yinchuan 750004, China

## Abstract

Peri-implant osteolysis (PIO) and the subsequent aseptic loosening are the main reasons for artificial joint implant failure. Existing methods for treating aseptic loosening are far from satisfactory, necessitating advanced drug exploration. This study is aimed at investigating the effect and underlying mechanism of tetrandrine (Tet) on inflammatory osteolysis. We established a Ti particle-induced inflammatory osteolysis mouse model and administered Tet or an equal volume of phosphate-buffered saline (PBS). Two weeks later, specimens were collected. Histological staining showed that Tet administration inhibited Ti-stimulated osteolysis. Tartrate-resistant acid phosphate (TRAP) staining and transmission electron microscopy (TEM) demonstrated that osteoclast formation was remarkably inhibited in the groups treated with Tet in a dose-dependent manner. In addition, relevant inflammatory cytokines (tumor necrosis factor (TNF)-*α*, interleukin (IL)-1*β*, and IL-6) were also significantly reduced in the calvaria of the Tet-treated groups. Exposure of receptor activator for nuclear factor-*κ*B ligand- (RANKL-) induced bone marrow-derived macrophages (BMMs) and RAW264.7 cells to Tet significantly reduced osteoclast formation, F-actin ring formation, bone resorption, and the expression of relevant genes (matrix metallopeptidase 9 (*MMP-9*), *TRAP*, and nuclear factor of activated T-cells, cytoplasmic 1 (*NFATc1*)) during osteoclastogenesis *in vitro*. Mechanistic studies using Western blotting demonstrated that Tet inhibited the nuclear factor (NF)-*κ*B signaling pathway by decreasing the phosphorylation of inhibitor of NF-*κ*B *α* (I*κ*B*α*) and p65, which play important roles in osteoclast formation. Collectively, our data indicate that Tet suppressed Ti-induced inflammatory osteolysis and osteoclast formation in mice, suggesting that Tet has the potential to be developed to treat and prevent wear particle-induced inflammatory osteolysis.

## 1. Introduction

Artificial joint replacement is recognized as an effective method for treating various end-stage joint diseases [1]. However, peri-implant osteolysis (PIO) is the major reason for prosthesis failure and remains unresolved. Abundant epidemiological evidence demonstrates that osteolysis and aseptic loosening around the prosthesis are predominantly attributable to the inflammation caused by the wear particles generated by repeated movement between prosthetic components [2–4]. Neutrophils are the first cells involved in the sterile inflammatory process, and they lead to the chronic activation of other phagocytes, creating a local cellular environment of cyclical acute inflammatory events.

Wozniak et al. [5] showed that the elevated levels of nitric oxide (NO) and other inflammatory factors produced by neutrophils may be important in the loosening of the joint prosthesis. Macrophages activated by wear particles also secrete various proinflammatory cytokines such as tumor necrosis factor (TNF)-*α*, interleukin (IL)-1*β*, and IL-6 [3, 6]. Recently, the concept of “osteoimmunology” has been recognized by many researchers [7]. The changes associated with this phenomenon induce the excessive activation of osteoclasts, which promote bone resorption and inhibit bone formation, ultimately leading to osteolysis and aseptic loosening around the prosthesis. Osteoclast maturation is regulated directly or indirectly by multiple cytokines and signaling pathways in the body, including the NF-*κ*B pathway, which is documented as the most significant [8].

Therefore, the activation of the NF-*κ*B signaling pathway is an important event in wear particle-induced osteolysis. Osteoclasts are multinucleated bone resorption cells derived from hematopoietic stem cells. Nuclear factor of activated T-cells, cytoplasmic 1 (NFATc1) is the main transcription factor necessary for osteoclast differentiation, and its expression and activation are regulated by the NF-*κ*B pathway [9]. Thus, decreasing the generation of osteoclasts by inhibiting NF-*κ*B signaling appears to be a feasible strategy for the treatment of PIO.

In addition to extensive research on aseptic loosening, drug-mediated prevention has been found to be promising in the treatment of PIO. Tet is a bisbenzylisoquinoline alkaloid extracted from the root of *Stephania tetrandra* S. Moore. Tet has a wide range of pharmacological effects and has been shown to be effective against silicosis, cancer, inflammation, and hypertension in clinical trials [10]. As an anti-inflammatory drug, Tet inhibits the activation of NF-*κ*B by inhibiting the degradation of I*κ*B*α*, thereby inhibiting the production of proinflammatory cytokines [11]. Tet is also a calcium channel blocker that can act on both L-type and T-type Ca^2+^ channels and has cardiovascular effects [12].

Several studies have proven that calcium channel blockers such as felodipine, cilnidipine, and benidipine can improve osteoporosis in ovariectomized mice by inhibiting osteoclasts [13–15]. A previous study by Takahashi et al. [16] showed that Tet alleviated bone loss in sciatic-neurectomized mice; however, this is a model of disused osteolysis. Jia et al. [17] reported that Tet plays an essential role in bone metabolism. Overall, these previous findings suggest that Tet may be an effective treatment agent for PIO caused by wear particles. However, the effectiveness of Tet against PIO has not yet been reported. Thus, in the present study, we aimed to explore the effect of Tet on wear particle-induced inflammatory osteolysis and the underlying molecular mechanisms.

## 2. Materials and Methods

### 2.1. Preparation of Ti Particles

**Figure 1:**
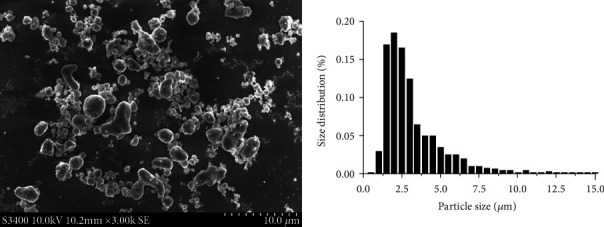
Scanning electron microscopy appearance of the Ti particles (magnification, 3000x).

Pure commercial Ti particles were obtained from Alfa Aesar Company (Ward Hill, MA, USA). A scanning electron microscope (Hitachi FESEM S-4800, Hitachi, Kyoto, Japan) was used to measure particle size, and more than 90% of particles were <10 *μ*m, which is the most common clinical size range (Figure 1). As previously described [18], Ti particles were washed three times with 70% ethanol solution for 48 h to remove the bound endotoxin. The particles were reconstituted in sterile phosphate-buffered saline (PBS) and diluted to 10 mg/mL. The absence of endotoxin was tested using a commercial Limulus assay kit (Chromogenic End-point TAL with a Diazo coupling kit, Xiamen Houshiji, Fujian, China). Particles with endotoxin levels <0.1 EU/mL were considered uncontaminated.

### 2.2. Ti Particle-Induced Mice Air Pouch Osteolysis Model

All animal-related experiments were conducted in accordance with the guidelines for the Care and Use of Laboratory Animals and were approved by the Animal Care Committee of NingXia Medical University (no: 2015--019). Sixty female-specific pathogen-free (SPF) BALB/c mice, 8–10 weeks old, weighing 22 ± 3 g, and in good health were selected. The animals were housed at five mice per cage in the SPF animal room of the Experimental Animal Center of Ningxia Medical University at room temperature (22 ± 2°C) and 60% relative humidity, under a 12 h light/dark cycle, and provided regular ad libitum access to food and water. The experiment was conducted after allowing acclimatization for 1 week.

No significant changes in the body weights of the mice were observed after modeling. Tet was obtained from Sigma-Aldrich (St. Louis, MO, USA). An air pouch osteolysis mouse model was established as previously described by Chen et al. [18, 19]. Briefly, 20 mice were selected and euthanized after anesthesia, and their skulls were immediately used as donor skulls for air pouch bone grafting in the other mice. The skull bone slice of one mouse can be used for air pouch bone grafting in two live mice. The remaining 40 mice were subjected to air pouch formation. On day 1, the mice were anesthetized (pentobarbital 50 mg/kg), and their backs were shaved, disinfected, and draped aseptically. Subsequently, 2 mL of air was injected to form an air pouch; 0.5 mL sterile air was subsequently injected into this pouch on days 2–6.

The air pouches were formed on day 7; next, 40 mice were randomly assigned to four groups of 10 animals each. In the first (sham) group, 0.5 mL PBS solution was injected into the air pouch, and 0.1 mL normal saline was injected intraperitoneally. In the second (vehicle) group, 0.5 mL Ti particle suspension (5 mg Ti) was injected into the air pouch, and 0.1 mL normal saline was injected intraperitoneally. In both the third and fourth groups, 0.5 mL Ti particle suspension (5 mg Ti) was injected into the air pouch, followed by 15 and 30 mg/kg Tet intraperitoneally (low- and high-Tet group, respectively). The dose of Tet used in this experiment was based on the manufacturer's instructions and was proven safe in a related study [20]. The injections were performed daily for 2 weeks after the bone and Ti implantation procedures; finally, all the mice were euthanized, and the pouch membranes with the intact bone implants were harvested for histological analysis.

### 2.3. Histological Analysis

To decalcify and fix the collected skull and membrane tissue, specimens were placed in a 12.5% ethylenediaminetetraacetic acid (EDTA) decalcification solution for 2 weeks. After dehydration and xylene-clearing treatment to render the tissue transparent, the specimens were paraffin-embedded, marked in groups, and stored at room temperature. Continuous 5 *μ*m tissue sections were sliced, transferred onto glass slides, baked, and then subjected to hematoxylin and eosin (H&E) and tartrate-resistant acid phosphate (TRAP) staining. TRAP staining was performed using a TRAP kit (Sigma-Aldrich, St. Louis, MO, USA) as described previously [21].

Images of the stained sections were examined using an Olympus DP70 microscope (Olympus Optical Co., Tokyo, Japan), and representative images were captured. The method established by Wooley et al. [22] was used to determine the thickness of the air pouch membrane and the eroded surface area. TRAP-positive cells were determined as the number of purple particles near the absorbed bone. The number and percentage of osteoclasts per bone surface (OcS/BS, %) were calculated according to the method proposed by Sawyer et al. [23].

The thickness of the pouch membrane and the number of TRAP-positive cells were evaluated using ImageJ software (National Institutes of Health (NIH), Bethesda, MD, USA). We collected the tissue on a copper mesh coated with a formvar film. Sections were mounted on glass slides, stained with methylene blue, and examined under a microscope. Ultrathin sections were prepared and collected on a copper mesh, followed by staining with 5% uranyl acetate in water for 4 min and lead citrate for 2 min. The sections were then observed under a transmission electron microscope (TEM, Hitachi H-7650, Hitachi, Kyoto, Japan).

For the immunohistochemical staining of TNF-*α*, IL-1*β*, and IL-6, paraffin-embedded sections were treated with 3% hydrogen peroxide added dropwise to inactivate endogenous peroxides and then heated in a microwave to perform antigen retrieval. A 5% goat serum was used to block the antigen for 10 min; subsequently, the sections were stained with rabbit anti-mouse primary antibodies (1 : 500; all purchased from Abcam, Cambridge, MA, USA) at 4°C for 8 h. After washing with PBS, the sections were incubated with goat anti-rabbit IgG (Proteintech Group, Chicago, IL, USA) for 30 min, and then, 3,3′-diaminobenzidine (DAB) dye solution was added dropwise to develop the color. The slides were counterstained with hematoxylin, dehydrated, made transparent, and mounted.

As negative (secondary antibody-only) controls, specimens were treated with PBS instead of the primary antibody to rule out nonspecific binding of the secondary antibody. All slides were independently examined by two experimenters using the Olympus DP70 microscope following the double-blind method, and the results were evaluated. Cells with brown particles in the cytoplasm were considered positive. At 40x magnification, five random fields of view on each slide were analyzed, and the image analysis software, ImageJ, was used to evaluate the positive expression rates of TNF-*α*, IL-1*β*, and IL-6 in each group.

### 2.4. Cell Culture and Osteoclast Differentiation

Mouse macrophages were isolated as previously described by Hu et al. [3] with a slight modification. Briefly, the mice were euthanized, and the bilateral femur and tibia were harvested and rinsed with PBS. After rinsing with the prepared medium and centrifugation, the cells were collected and incubated in Dulbecco's modified Eagle's medium (DMEM, Gibco, Thermo Fisher Scientific, Inc., Waltham, MA, USA) containing 10% fetal bovine serum (Gibco, Thermo Fisher Scientific, Inc.), 100 IU/mL penicillin, 100 *μ*g/mL streptomycin (Beijing Solarbio Science and Technology Co., Ltd., Beijing, China), and 50 ng/mL macrophage colony-stimulating factor (M-CSF, R&D Systems, Minneapolis, MN, USA) for 12 h. Unadhered cells were harvested and cultured in six-well plates in a 37°C/5% CO_2_ incubator.

After overnight incubation, the adherent cells were considered BMMs. These cells were then transplanted into 96-well plates at a density of 8 × 10^3^ cells/well and pretreated with or without Tet (0.0, 0.1, 0.5, and 1.0 *μ*M) for 4 h. To induce osteoclast differentiation, 50 ng/mL M-CSF and 100 ng/mL RANKL (R&D Systems, Minneapolis, MN, USA) were added to the medium, and the cells were cultured for an additional 5 days with a change of medium every 3 days. To determine the stage (early or late) at which Tet affects osteoclast formation, 1.0 *μ*M Tet was added to the induction medium on days 0, 1, 2, or 3, and TRAP staining was performed on day 5. The cell culture medium was changed every 3 days in this experiment. TRAP-positive multinucleated cells (three or more nuclei) were counted under the Olympus DP70 microscope.

### 2.5. Cell Viability Assay

RAW264.7 macrophages were obtained from the Type Culture Collection of the Chinese Academy of Sciences (Shanghai, China). To ascertain that the Tet dose used in this experiment had no toxicity on BMMs and RAW264.7 cells, a cell viability assay was performed using cell counting kit-8 (CCK-8, Jiangsu KeyGen Biotech Co., Ltd., Nanjing, China). BMMs (5 × 10^3^ cells/well) and RAW264.7 cells (3 × 10^3^ cells/well) were seeded in a 96-well plate, incubated with M-CSF (50 ng/mL) and RANKL (100 ng/mL), and then treated with the indicated doses of Tet (0, 0.1, 0.3, 0.5, 0.7, 1.0, 5.0, and 10.0 *μ*M) for 72 h. Then, 10 *μ*L of a CCK-8 assay solution was added to each well, and the cells were incubated for an additional 2 h. The absorbance was measured at 450 nm using a E2500 microplate reader (Thermo Fisher Scientific, Inc.).

### 2.6. F-Actin Ring Staining Assay

BMMs (8 × 10^3^ cells/well) were cultured in a 96-well plate as described in the previous section, fixed with 4% paraformaldehyde, and permeabilized with 0.1% (*v*/*v*) Triton X-100. After extensive rinsing with PBS, the cells were incubated with Alexa-Fluor 647 phalloidin (Invitrogen, San Diego, CA, USA) for 1 h. Subsequently, the BMMs were rinsed three times with PBS, the nuclei were counterstained with 4′,6-diamidino-2-phenylindole (DAPI), and the stained cells were examined under a confocal microscope (Carl Zeiss, Oberkochen, Germany).

### 2.7. Resorption Pit Formation Assay

BMMs (2.4 × 10^4^ cells/well) were plated in hydroxyapatite-coated six-well plates (Corning Inc., NY, USA). The cells were allowed to adhere to the wells for a few hours to overnight; they were then pretreated with various Tet concentrations in basal medium for 4 h. To induce osteoclast differentiation, RANKL (100 ng/mL) and M-CSF (50 ng/mL) were added to the medium, and the cells were cultured for an additional 5 days with a change of medium every 3 days. Sonication was used to remove the cells attached to the bottom of the wells. Resorption was observed under the DP70 microscope, and the resorption pit area was measured using ImageJ software.

### 2.8. Enzyme-Linked Immunosorbent Assay (ELISA)

RAW264.7 cells (1 × 10^4^ cells/well) were plated in six-well plates and divided into the following five groups that were treated as indicated: sham, cultured in DMEM; vehicle, 0.1 mg/mL Ti particles; and low-, medium-, and high-dose Tet, each treated with 0.1 mg/mL Ti particle suspension, followed by 0.1, 0.5, and 1.0 *μ*M Tet, respectively; subsequently, all the groups were cultured for 5 days with a change of medium every day. The culture supernatant was collected on days 1–5 and stored at -80°C. ELISA was performed using specific kits for TNF-*α* and MMP-9 (all purchased from eBioscience, San Diego, CA, USA) according to the manufacturer's instructions. Optical density was measured at 450 nm using a microplate reader (Thermo Fisher Scientific, Inc.).

### 2.9. Gene Expression of *TRAP*, *MMP-9*, and *NFATc1*

The various groups of RAW274.7 cells were cultivated for 5 days and then lysed; total RNA was extracted using TRIzol reagent (Invitrogen). Complementary cDNA was synthesized from 1 *μ*g total RNA using a RevertAid First Strand cDNA synthesis kit (Thermo Fisher Scientific, Inc.). Reverse transcription-polymerase chain reaction (RT-PCR) was performed using a LightCycer PCR system (Roche, Switzerland) with the SYBR Premix Ex Tag kit (TaKaRa, Japan) in 10 *μ*L RT-PCR reaction buffer. After denaturation at 95°C for 5 min, PCR was performed with 40 cycles of denaturation at 95°C for 10 s, annealing at 60°C for 15 s, and extension at 72°C for 10 s. All experiments were performed in triplicate, and *β*-actin was used to normalize the target gene levels. The primer sequences used were as follows: *β*-actin, forward 5′-AGGGTGTGATGGTGGGAATG-3′ and reverse 5′-GCTGGGGTGTTGAAGGTCTC-3′; TRAP, forward 5′-AGGGTGTGATGGTGGGAATG-3′ and reverse 5′-GCTGGGGTGTTGAAGGTCTC-3′; MMP-9, forward 5′-GCTGAAACCAGACCCCAGAC-3′ and reverse 5′-TGACCTGAACCATAACGCACA-3′; and NFATc1, forward 5′-CCAATGAGCCAGGGGATTAG-3′ and reverse 5′-GCAGGAGAGGAAAGGTCGTG-3′. The relative expression of the target cellular mRNA was calculated using the 2^−ΔΔCt^ method.

### 2.10. Western Blot Assay

Western blotting was performed to determine the possible mechanisms mediating the inhibitory effects of Tet on osteoclastogenesis. The expression levels of I*κ*B*α*, p-I*κ*B*α*, NF-*κ*B p65, p-NF-*κ*B p65, and *β*-actin were detected using the following steps: (1) RAW264.7 cells were seeded in 6-well plates at a density of 6 × 10^5^ cells/well until fully grown; they were then pretreated with or without 1.0 *μ*M Tet for 4 h. Subsequently, 100 ng/mL RANKL was added to stimulate the cells for 0, 15, 30, or 60 min. (2) For the ELISA of the treatment group samples, RAW 274.7 cells were cultured for 5 days and collected; they were then treated with 50 *μ*L radioimmunoprecipitation assay (RIPA) lysis buffer (Nanjing KeyGen Biotech Co., Ltd., Nanjing, China), containing proteinase and phosphatase inhibitors.

Protein samples (30 *μ*g) were separated using 10% sodium dodecyl sulfate (SDS)-polyacrylamide gel electrophoresis and transferred to polyvinylidene fluoride (PVDF) membranes; the membranes were blocked by incubating in 5% skim milk in Tris-buffered saline (TBS)-Tween (TBS-T, 10 mM Tris-HCl, 50 mM sodium chloride (NaCl), 0.25% Tween 20) for 1 h. The membrane was incubated overnight at 4°C with primary antibodies (I*κ*B*α*, p-I*κ*B*α*, NF-*κ*B p65, p-NF-*κ*B p65, all 1 : 1000; and *β*-actin, 1 : 2000; Cell Signaling Technology). After three rinses with TBS-T, the membranes were incubated with horseradish peroxidase (HRP) anti-rabbit secondary antibody (1 : 5000; Proteintech) for 1 h. The signals were detected via exposure in a Bio-Rad imaging system. Gray levels corresponding to the indicated proteins were quantified and normalized relative to *β*-actin using ImageJ software.

### 2.11. Statistical Evaluation

Data processing and statistical analysis were performed using the statistical package for the social sciences (SPSS) 22.0 (IBM Corp., Armonk, NY, USA). Data are expressed as the mean ± standard error. Measured data were analyzed using analysis of variance, and an independent sample *t*-test was used to compare the groups, followed by Student's *t*-test (comparing two groups) or the least significant difference post hoc test (comparing more than two groups). Differences were considered statistically significant at ^∗^*p* < 0.05 or ^∗∗^*p* < 0.01.

## 3. Results

### 3.1. Tet Ameliorate Ti Particle-Induced Inflammatory Osteolysis *In Vivo*

**Figure 2:**
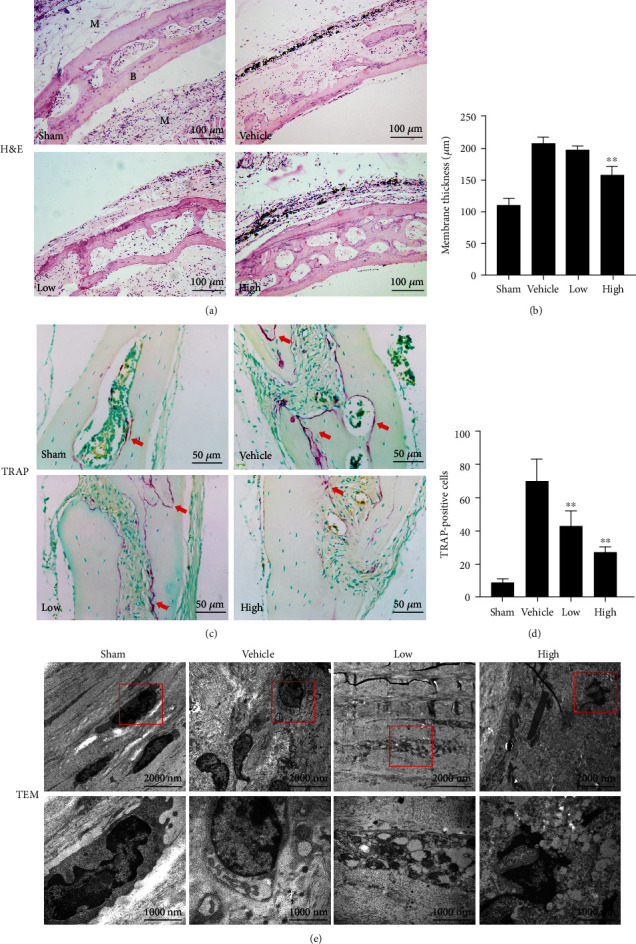
Tetrandrine (Tet) prevents Ti particle-induced chronic inflammation and osteolysis *in vivo*. (a) H&E-stained images of the tissue specimen sections in each group observed via light microscopy. (b) Membrane thickness were quantified. (c) TRAP-stained images of the tissue specimen sections in each group observed via light microscopy. (d) TRAP-positive cells were quantified. (e) Transmission electron microscopy (TEM) images of cells in the implanted bones and pouches around Ti particles. The air pouch membrane thickness (*μ*m) and TRAP-positive cell numbers were quantified (^∗^*p* < 0.05, ^∗∗^*p* < 0.01 compared with the vehicle group; *n* = 5 per group). M: membrane; B: bone; H&E: hematoxylin and eosin; TRAP: tartrate-resistant acid phosphatase. The red arrows indicate TRAP-positive cells.

To investigate the effect of Tet on Ti particle-induced inflammatory osteolysis, we created air pouches on the backs of BALB/c mice and embedded cranial bone allografts inside; this was followed by the injection of Ti particles. As shown in Figure 2(a), Ti stimulation obviously induced inflammatory reactions and bone erosion, whereas Tet administration significantly suppressed this inflammation and bone destruction in a dose-dependent manner. This was evidenced by the thinner air pouch membranes and fewer erosion pits in the bones of the Tet-treated groups than in those of the vehicle group (Figures 2(a) and 2(b)). In addition, TRAP staining showed a remarkably higher number of osteoclasts on the bone surface of the Ti-treated group than on that of the sham group. However, the number of osteoclasts was significantly decreased following Tet treatment (Figures 2(c) and 2(d)).

TEM was used to observe the ultrastructure of the tissues. As shown in Figure 2(e), the sham group osteoclasts exhibited a fine shape, clear nucleoli, fine chromatin particles, uniform distribution, clear cell boundaries, and few lysosomes, whereas the vehicle group showed multiple active osteoclasts, characterized by a large cell size, rich organelles, numerous rough endoplasmic reticulum and mitochondria in the cytoplasm, and an obvious increase in lysosomes. In contrast, the Tet-treated group showed fewer osteoclasts in the bone tissue than the vehicle group, and the osteoclasts exhibited fewer organelles, disorderly arrangement, swollen mitochondria, blurred mitochondrial cristae, damaged mitochondria, and serious cavitation. These results indicate that Tet effectively reduce the inflammatory bone erosion induced by Ti particles *in vivo*, which may be attributed to its inhibitory effect on osteoclast formation.

### 3.2. Tet Inhibits the Release of Inflammatory Cytokines Associated with Osteolysis *In Vivo*

**Figure 3:**
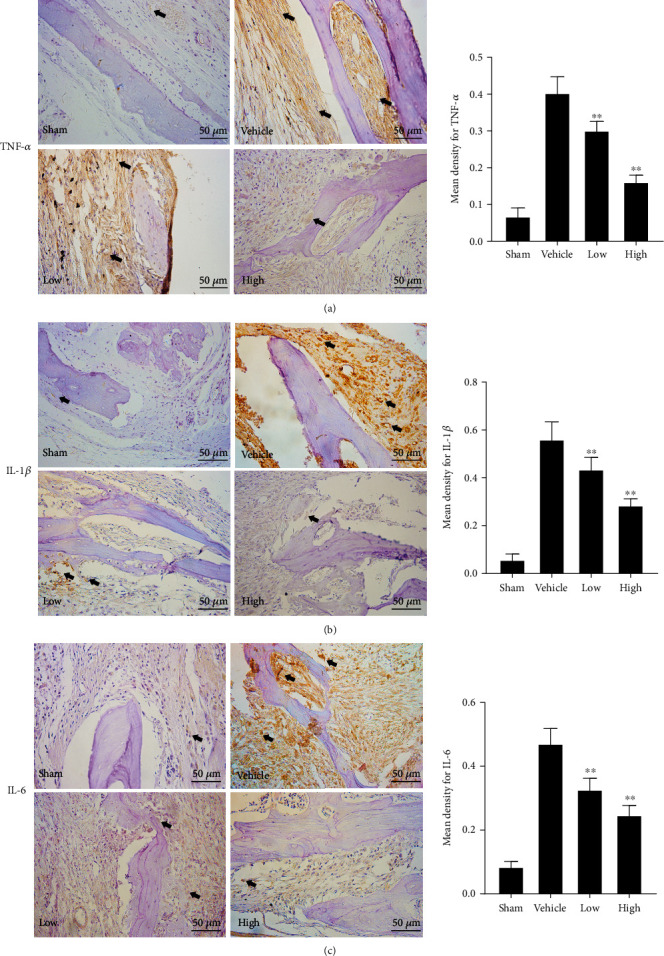
Tetrandrine (Tet) inhibits the expression of proinflammatory cytokines. (a) Immunohistochemical staining of tumor necrosis factor (TNF)-*α*. (b) Immunohistochemical staining of interleukin (IL)-1*β*. (c) Immunohistochemical staining of IL-6. The mean density for TNF-*α*, IL-1*β*, and IL-6 were quantified (^∗^*p* < 0.05, ^∗∗^*p* < 0.01 compared with the vehicle group; *n* = 5 per group). Black arrows indicate positive expression, respectively.

Proinflammatory cytokines are believed to contribute to bone resorption through the promotion of osteoclast differentiation [24, 25]. Thus, we evaluated the expression of the following cytokines associated with osteoclastogenesis: TNF-*α*, IL-1*β*, and IL-6. Immunohistochemical analysis revealed that Ti particles drastically increased the expression of TNF-*α*, IL-1*β*, and IL-6 in the bone-graft samples (Figures 3(a)–3(c)). However, Tet treatment significantly and dose-dependently inhibited the expression of TNF-*α*, IL-1*β*, and IL-6. These data demonstrate that Tet inhibits the secretion of proinflammatory cytokines induced by Ti particles *in vivo*.

### 3.3. Tet Inhibits RANKL-Induced Osteoclast Formation without Cytotoxicity *In Vitro*

**Figure 4:**
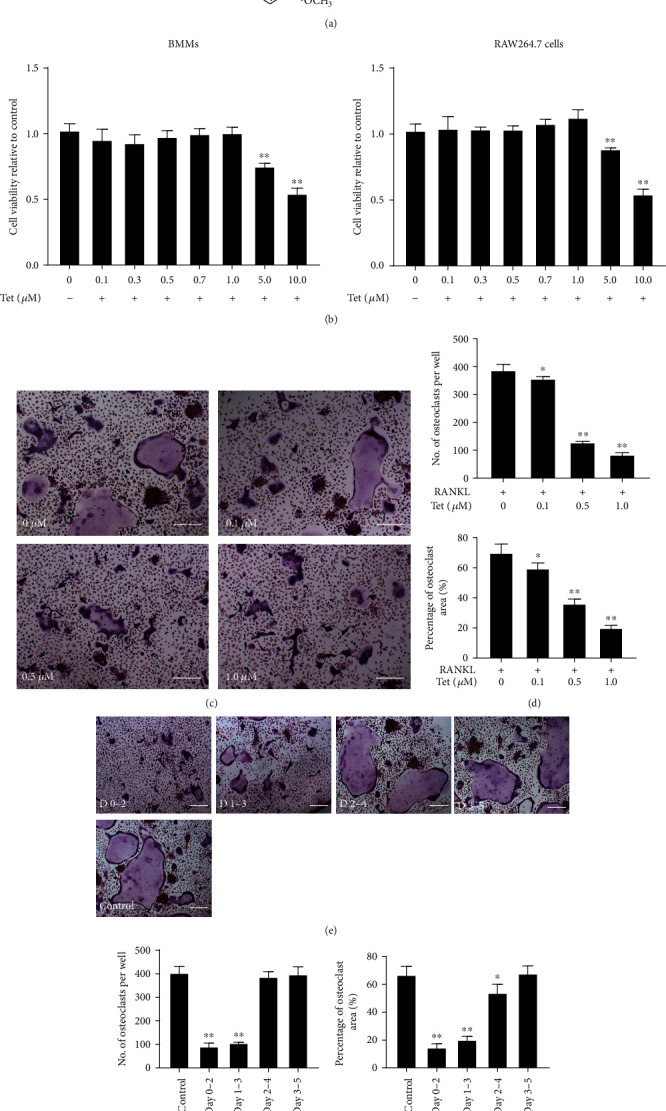
Tetrandrine (Tet) inhibits receptor activator for nuclear factor-*κ*B ligand- (RANKL-) induced osteoclast formation and is not cytotoxic. (a) Chemical structure of Tet. (b) Bone marrow-derived macrophages (BMMs) and RAW264.7 cell viabilities were detected using the cell counting kit-8 (CCK-8) assay, and the results were normalized to the control group (without Tet treatment). (c) BMMs were stimulated with the indicated concentrations of Tet in osteoclast differentiation medium and then fixed and subjected to TRAP staining on day 5 (scale bar = 100 *μ*m). (d) The number and area of osteoclasts. (e) BMMs were incubated in Dulbecco's modified Eagle's medium (DMEM) supplemented with M-CSF and RANKL, with Tet (1.0 *μ*M) administered on days 0–2, 1–3, 2–4, or 3–5, and TRAP staining was performed to analyze osteoclast formation (scale bar = 100 *μ*m). (f) The number and area of osteoclasts. All experiments were repeated three times (^∗^*p* < 0.05, ^∗∗^*p* < 0.01 compared with the control group; *n* = 5 per group). Data are the means of at least three independent experiments with similar results. TRAP: tartrate-resistant acid phosphatase; BMMs: bone marrow-derived macrophages; M-CSF: macrophage colony-stimulating factor.

To determine the effect of Tet on osteoclastogenesis, primary BMMs and the osteoclast precursor RAW264.7 cell line were cultured *in vitro.* We first performed a CCK-8 assay to evaluate the cytotoxicity of Tet and then confirmed the safe concentration for the *in vitro* experiments. The results showed that the viability of BMMs and RAW264.7 cells was not affected by Tet at concentrations <1.0 *μ*M (Figure 4(b)). Then, RANKL-induced BMMs were treated with different concentrations of Tet (0, 0.1, 0.5, and 1.0 *μ*M) or an equal volume of vehicle for 5 days. We observed that Tet inhibited RANKL-stimulated osteoclast formation in a dose-dependent manner, as indicated by the significantly lower number of TRAP-positive osteoclasts in the Tet-treated groups than in the vehicle group (Figures 4(c) and 4(d)).

To further clarify the stage of the osteoclast formation process affected by Tet, RANKL-induced BMMs were treated with 1.0 *μ*M Tet at different time points (days 0–2, 1–3, 2–4, or days 3–5), and vehicle-treated BMMs were used as the control. The results demonstrated that both the number and total area of Tet-treated osteoclasts were significantly smaller than those of the control in the early periods (days 0–2 and days 1–3). However, no obvious difference was observed with the Tet-treated cells in the later periods (days 2–4 or 3–5) (Figures 4(e) and 4(f)). These results suggest that Tet inhibited RANKL-induced osteoclast formation in the early stage *in vitro.*

### 3.4. Osteoclastic F-Actin Ring Formation and Bone Resorption Shows That Tet Inhibits Bone Resorption *In Vitro*

**Figure 5:**
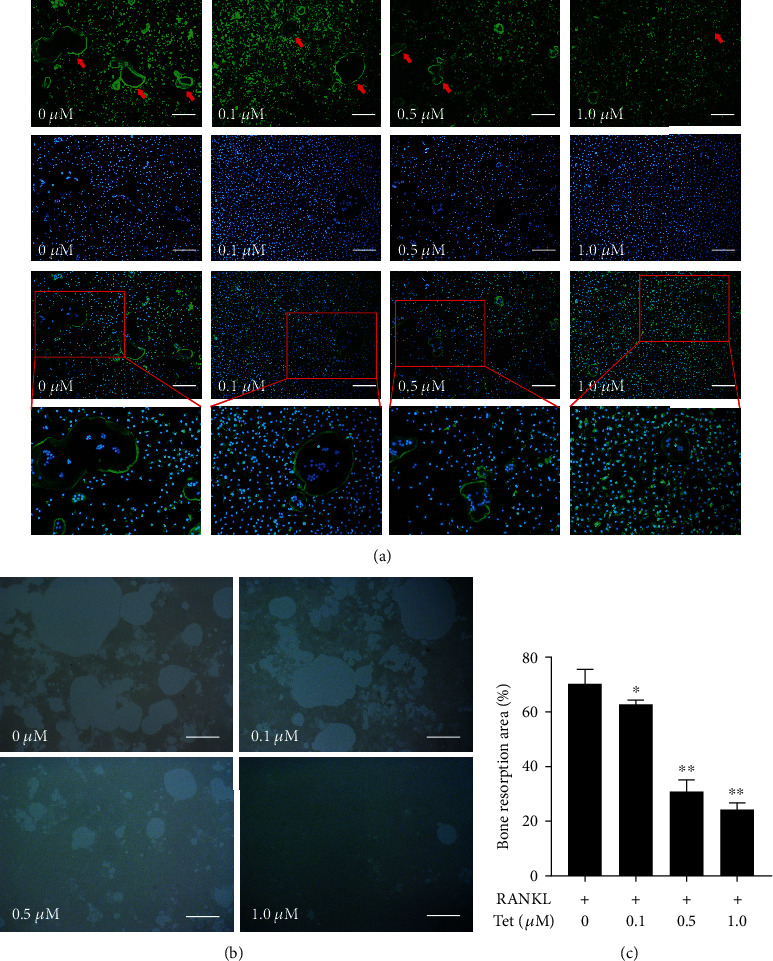
Tetrandrine (Tet) decreases osteoclast bone resorption and the formation of F-actin rings. (a) F-actin rings and nuclei were observed under a confocal microscope (scale bar = 100 *μ*m). (b) Images of the bone resorption pits in each group (scale bar = 100 *μ*m). (c) Quantification of bone resorption area (^∗^*p* < 0.05, ^∗∗^*p* < 0.01 compared with the control group; *n* = 4 per group). Data are the means of at least three independent experiments with similar results. The red arrows indicate the F-actin rings.

The actin cytoskeleton is a decisive structure for the maturation of functional osteoclasts [26]. In line with the results above, treatment with Tet remarkably reduced the size of the F-actin ring in RANKL-stimulated BMMs in a dose-dependent manner (Figure 5(a)). We further verified the effect of Tet on the bone resorption ability of osteoclasts by culturing BMMs in the wells of plates coated with hydroxyapatite. The sizes of the resorption pits were calculated to evaluate the resorption ability of the osteoclasts. As expected, the resorption area was significantly smaller in the groups exposed to Tet than in the control groups, and the effect was dependent on the Tet concentration (Figures 5(b) and 5(c)), indicating that Tet impaired osteoclast bone resorption. These findings demonstrate that Tet decreased osteoclast formation and subsequent bone resorption *in vitro*.

### 3.5. Tet Suppresses the Expression of NFATc1, TRAP, TNF-*α*, and MMP-9 in Osteoclasts

**Figure 6:**
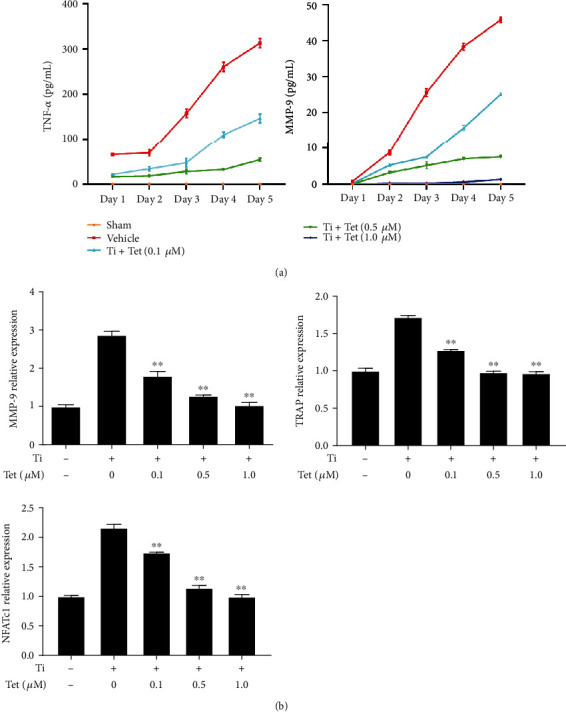
Tetrandrine (Tet) reduces the expression of osteoclast-related genes (including nuclear factor of activated T-cells, cytoplasmic 1 (NFATc1), tartrate-resistant acid phosphatase (TRAP), tumor necrosis factor (TNF)-*α*, and matrix metallopeptidase (MMP)-9). (a) TNF-*α* and MMP-9 secretion in the supernatant of the RAW264.7 cell culture medium in each group on days 1–5. (b) *MMP-9*, *TRAP*, and *NFATc1* mRNA expression in each group on day 5 (^∗^*p* < 0.05, ^∗∗^*p* < 0.01 compared with the control group; *n* = 3 per group). Data are the means of at least three independent experiments with similar results.

To explore the effect of Tet on the expression of osteoclastogenesis-related proteins, we collected the supernatants of Ti particle-stimulated RAW264.7 cells treated with different concentrations of Tet or the equivalent vehicle daily from days 1 to 5. Cells cultured without Ti particles and Tet were used as the sham group. An ELISA was performed to determine the time course of the secretion of TNF-*α* and MMP-9, which are involved in osteoclast formation [27]. The results showed that Tet dramatically inhibited TNF-*α* and MMP-9 secretion in the supernatants at all time points, in a dose-dependent manner (Figure 6(a)).

We further examined the expression of MMP-9, TRAP, and NFATc1, which play a critical role in osteoclast maturation and bone resorption [28, 29]. The real-time PCR showed that the expression of *MMP-9*, *TRAP*, and *NFATc1* was markedly increased in RAW264.7 cells stimulated with Ti particles, whereas Tet treatment significantly reduced the expression of these genes (Figure 6(b)), which was consistent with the results showing decreased osteoclast formation and bone resorption ability. Thus, these results confirmed the inhibitory effect of Tet on osteoclast differentiation and bone resorption *in vitro*.

### 3.6. Tet Inhibits the RANKL-Induced Activation of the NF-*κ*B Signaling Pathway

**Figure 7:**
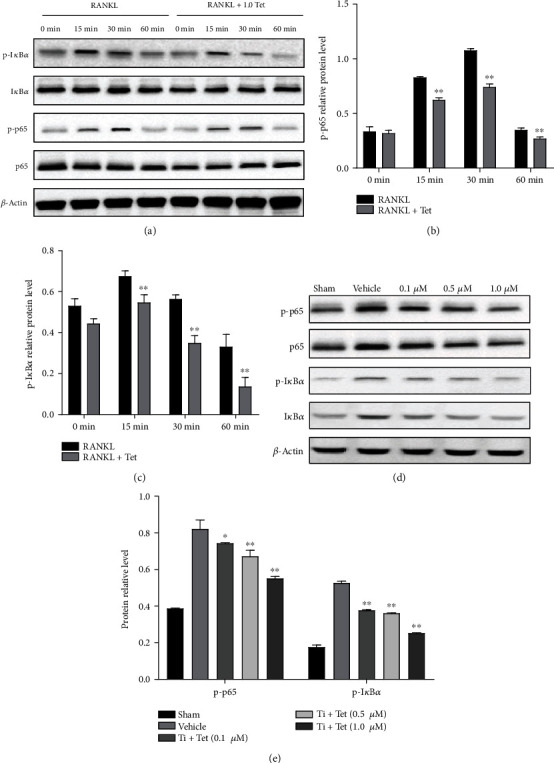
Tetrandrine (Tet) inhibits osteoclastogenesis through the nuclear factor (NF)-*κ*B signaling pathway. (a) Protein expression of the NF-*κ*B signaling pathway. (b, c) Gray band levels of the phosphorylated inhibitor of NF-*κ*B *α* (p-I*κ*B*α*) (b) and phosphorylated p-65 (p-p65) (c) were analyzed using Image J software. (d) After 5 days of treatment with various Tet concentrations, the cells were lysed for Western blotting, and the gray band levels of p-p65 and I*κ*B*α* were analyzed using ImageJ software (^∗^*p* < 0.05, ^∗∗^*p* < 0.01 compared with the vehicle group; *n* = 3 per group). Data are the means of at least three independent experiments with similar results.

To further elucidate the mechanism underlying the Tet-induced inhibition of osteoclasts, Western blot analysis was conducted to determine its effect on the activation of NF-*κ*B signaling, which is a key regulator of the differentiation and activity of osteoclasts [30]. We examined the phosphorylation levels of the main NF-*κ*B subunits, I*κ*B*α* and p65, in RANKL-induced RAW264.7 cells treated with or without 1.0 *μ*M Tet at four time points (0, 15, 30, and 60 min). Unsurprisingly, RANKL stimulation obviously induced the phosphorylation of I*κ*B and p65; however, the expression levels of phosphorylated-I*κ*B*α* (p-I*κ*B*α*) and p-p65 were significantly decreased in the cells exposed to Tet (Figures 7(a)–7(c)), indicating that Tet suppressed the RANKL-induced activation of the NF-*κ*B signaling pathway. To further verify the effect of Tet on the NF-*κ*B pathway, the expression of related proteins was examined in RAW264.7 cells stimulated with Ti and different concentrations of Tet or the vehicle. As shown in Figures 4(d) and 4(e), the expression levels of p-I*κ*B and p-p65 were negatively correlated with the concentrations of Tet ranging from 0.1 to 1.0 *μ*M. Therefore, collectively, these and the previous results led us to conclude that Tet may hinder the differentiation and maturation of osteoclasts by inhibiting the NF-*κ*B signaling pathway.

## 4. Discussion

Artificial aseptic loosening is one of the main reasons for revision surgery after artificial joint replacement. Aseptic loosening is a process involving complex interplay between mechanical factors, such as poor matching, improper positioning, or aging of the prosthesis, and stress shielding [31]. Recent studies have shown that long-term friction causes the implanted prosthesis to produce tiny particles, which induce the release of inflammatory factors and the activation of osteoclasts, which play an essential role in the osteolysis around the prosthesis [32, 33].

Previously, micron-sized particles were considered the main particles that induce osteolysis, but the introduction of laser capture microdissection technology and TEM has led to the detection of various nanosized particles in loosened boundary membrane tissues, including metal, ceramic granular, and polymer polyethylene nanoparticles. Because nanoparticles are more easily engulfed by phagocytes than other particles, they may play a considerably significant role in aseptic loosening. These particles activate NF-*κ*B, mitogen-activated protein kinase (MAPK), and other signaling pathways, initiating the transcriptional expression of inflammatory factors such as TNF-*α*, IL-1*β*, and IL-6 directly or indirectly. These factors induce the differentiation and maturation of osteoclasts and enhance their activity [34].

Presently, progress has been made in studies using bisphosphonate drugs and gene therapy to inhibit the adverse reactions of wear particles, but considerable modifications are still required before their clinical application. In this study, we established an inflammatory osteolysis mouse model by transplanting cranium grafts into air pouches created in recipient mice, followed by Ti particle stimulation. Pathological changes similar to inflammatory osteolysis were observed, such as increased cellular infiltration and bone erosion. Tet treatment dramatically alleviated the inflammatory reaction and bone destruction, suggesting that it may be a potential agent for the prevention and treatment of osteolysis in artificial aseptic loosening. Although the air pouch osteolysis model is frequently used in wear debris-induced osteolysis studies, they have several limitations, which should not be ignored. These include the lack of blood supply to the bone graft and the use of bony surfaces, which are not typically involved in artificial aseptic loosening. It would be better to use a combination of different osteolysis models in the study, such as a calvarial osteolysis model where osteolysis is directly induced in the skull of animals, which we are considering for our future studies.

Recently, traditional herbs and extracts have been receiving increasing attention for the treatment of aseptic loosening of prostheses caused by osteolysis. Tet is a natural substance with a wide range of pharmacological effects, including antifibrosis, antitumor, and anti-inflammatory effects [10, 35–37]. Numerous studies have reported that Tet exerts anti-inflammatory effects through a variety of signaling pathways such as the NF-*κ*B, phosphoinositide 3-kinase (PI3K), and extracellular signal-regulated kinase (ERK), signal transducer and activator of transcription 3 (STAT3) signaling pathways [11, 38–40]. Guo et al. [41] found that aqueous extracts and alkaloids of *S. tetrandra* inhibited the release of NO, TNF-*α*, and IL-6, alleviating the LPS-induced inflammatory response in RAW264.7 cells.

NF-*κ*B is a multifunctional transcription factor that plays a critical role in both inflammation and osteoclast formation [42], suggesting that strategies targeting NF-*κ*B inhibition may be promising for the treatment of inflammatory osteolysis. In the present study, we found that Tet suppressed the activation of the NF-*κ*B pathway and relieved both inflammatory reactions and osteoclast formation in mice, indicating that it exerts therapeutic effects via dual functions of hindering inflammation and osteoclastogenesis.

Tet is also known to be a calcium channel blocker. Calcium (Ca^2+^) signaling is important for multiple osteoclast functions, including gene transcription, differentiation, and bone resorption [43]. RANKL-induced Ca^2+^ oscillation mediates the activation of NFATc1, which is an essential transcription factor for osteoclast differentiation [44]. In this study, Tet administration significantly inhibited osteoclast formation and the mRNA expression of NFATc1 *in vitro*, suggesting that in addition to inhibiting the NF-*κ*B pathway, Tet may also hamper osteoclast formation by affecting Ca^2+^ signaling. However, we have not examined the role of Tet in Ca^2+^ signaling during osteoclast differentiation. We also cannot exclude the possible involvement of other mechanisms in the effect of Tet on osteoclastogenesis, which warrants further investigation.

We did not examine the toxicity of Tet in animals. Tet has been reported to induce transient toxicity in the kidneys, lungs, and liver of mice following intravenous injection of a single 150 mg/kg dose [45]. However, in this study, no obvious abnormal conditions were observed in mice intraperitoneally administered a single 30 mg/kg dose of Tet. In addition, a clinical study of the efficacy of Tet in patients with lung cancer reported no side effects [46]. Thus, Tet may be considered a safe candidate for clinical application in the treatment of inflammatory osteolysis, which warrants further examination.

## 5. Conclusions

The findings of this study demonstrated that Tet effectively reduced the inflammation and bone resorption induced by Ti particles *in vivo* and inhibited osteoclast differentiation and formation *in vitro* through the inhibition of NF-*κ*B signaling. Our study offers novel insights into the mechanism of the established anti-inflammatory and antibone resorption effects of Tet, providing an evidence-based rationale for its potential for use in the treatment of osteolysis developing around implants.

## Data Availability

The data used to support the findings of this study are available from the corresponding author upon reasonable request: Desheng Chen: chendesheng@nxmu.edu.cn.
